# Stoichio-Metagenomics of Ocean Waters: A Molecular Evolution Approach to Trace the Dynamics of Nitrogen Conservation in Natural Communities

**DOI:** 10.3389/fmicb.2018.01590

**Published:** 2018-07-18

**Authors:** Hannes Dittberner, Niklas Ohlmann, Claudia Acquisti

**Affiliations:** Institute for Evolution and Biodiversity, University of Münster, Münster, Germany

**Keywords:** stoichiogenomics, nitrogen limitation, marine microbial communities, molecular evolution, material costs

## Abstract

Nitrogen is crucially limiting in ocean surface waters, and its availability varies substantially with coastal regions typically richer in nutrients than open oceans. In a biological stoichiometry framework, a parsimonious strategy of nitrogen allocation predicts nitrogen content of proteins to be lower in communities adapted to open ocean than to coastal regions. To test this hypothesis we have directly interrogated marine microbial communities, using a series of metagenomics datasets with a broad geographical distribution from the Global Ocean Sampling Expedition. Analyzing over 20 million proteins, we document a ubiquitous signal of nitrogen conservation in open ocean communities, both in membrane and non-membrane proteins. Efficient nitrogen allocation is expected to specifically target proteins that are expressed at high rate in response to nitrogen starvation. Furthermore, in order to preserve protein functional efficiency, economic nitrogen allocation is predicted to target primarily the least functionally constrained regions of proteins. Contrasting the NtcA-induced pathway, typically up-regulated in response to nitrogen starvation, with the arginine anabolic pathway, which is instead up-regulated in response to nitrogen abundance, we show how both these predictions are fulfilled. Using evolutionary rates as an informative proxy of functional constraints, we show that variation in nitrogen allocation between open ocean and coastal communities is primarily localized in the least functionally constrained regions of the genes triggered by NtcA. As expected, such a pattern is not detectable in the genes involved in the arginine anabolic pathway. These results directly link environmental nitrogen availability to different adaptive strategies of genome evolution, and emphasize the relevance of the material costs of evolutionary change in natural ecosystems.

## Introduction

Recent investigations in “stoichiogenomics,” projecting biological stoichiometry into molecular evolution, have indicated that the atomic composition of proteins and genes has adaptive significance (reviewed in Elser et al., 2011). These studies have explored the material cost of evolutionary change, showing how environmental nutrient limitations have affected the composition of the genetic material (Baudouin-Cornu et al., 2004; Bragg and Hyder, 2004; Bragg et al., 2006; Elser et al., 2006; Bragg and Wagner, 2007, 2009; Acquisti et al., 2009a,b; Gilbert and Fagan, 2011; Read et al., 2017). In particular, nitrogen limitation has been the focus of many of these analyses because nitrogen is an essential component of amino acids and nucleotides, and it is often limiting in natural environments. The impact of selection for nitrogen conservation in shaping evolutionary change remains, however, elusive. One of the problems is that previous analyses have primarily relied on model organisms, leaving the relevance of adaptation to nutrient availability in natural environments only partially addressed. The aim of this paper is to bridge this gap, and to directly quantify the role of selection for nitrogen conservation in a natural ecosystem, combining the power of metagenomics and biogeochemistry in an evolutionary framework.

Covering almost three quarters of the Earth’s surface and more than half of the planetary total net primary productivity, oceans play a profound role in tuning the global dynamics of nutrient pools in the biosphere. In the complex regulation of the oceanic bio-geochemical cycles, the severe depletion of inorganic nitrogen in surface waters is one of the factors critically limiting growth and reproduction in seawaters (Capone and Hutchins, 2013). Nutrient regimes are however highly variable in oceans. For example, the severity of nitrogen limitation in surface waters follows a clear spatial pattern, as it decreases dramatically in coastal regions due to upwelling, and to terrestrial and riverine nutrient inputs (Capone and Hutchins, 2013). In the last decades this effect has been on the rise, as nutrient enrichment of coastal zones has substantially increased due to anthropogenic activity. Due to the intrinsic variation of nitrogen availability between coastal and open ocean surface waters, the different marine biota adapted to these two habitats provide an ideal set of related ecosystems to study the effect of environmental nitrogen limitation on the evolution of proteins.

The Global Ocean Sampling Expedition (Rusch et al., 2007; Yooseph et al., 2007), a comprehensive catalog of marine surface water metagenomics sampling across the globe, offers a useful dataset to address the role of nitrogen limitation in shaping evolutionary change in marine microbial communities. Under persistent conditions of severe nitrogen limitation, growth and reproduction of individuals with lower allocation of nitrogen in their proteins should be favored by natural selection. Thus, species adapted to oligotrophic open ocean waters are expected to exhibit a progressive evolutionary shift in the frequencies of amino acids toward an enrichment of amino acids with a lower relative concentration of nitrogen. Indeed a previous analysis (Grzymski and Dussaq, 2012) has shown that the average nitrogen content of proteins is significantly lower in open ocean than in coastal microbial communities.

Merging the perspective of molecular evolution and biological stoichiometry, we say that selection for the fittest is expected to shape nitrogen allocation while preserving protein structure and function. However, protein structural constraints alone strongly affect amino acid composition, and consequently protein nitrogen content. This is independent from adaptation to environmental nitrogen scarcity. As such, it is compelling to tell apart the contribution of these two forces in shaping protein nitrogen content. To shed light on these critical points, here we take an evolutionary approach to analyze the dynamics of nitrogen allocation in different functional and structural classes of proteins in natural communities.

Membrane proteins, in order to be inserted into the lipidic cellular membranes, are bound to be rich in hydrophobic amino acids. Due to the specific stoichiometry of hydrophobic amino acids (Berg et al., 2002), this structural constraint alone leads to a low nitrogen content. Given that membrane proteins typically cover 12% of marine metagenomes (Patel et al., 2010), their weight in shaping nitrogen allocation in natural communities is particularly relevant. In order to address this important aspect, we have identified (Käll et al., 2004) all membrane proteins in the dataset, and studied nitrogen allocation separately in membrane and non-membrane proteins. We have found that protein nitrogen content is lower in communities adapted to oligotrophic open oceans in both membrane and non-membrane proteins. Membrane proteins are further shaped by structural constraints that result in a sharp distribution of charged residues between the two sides of cellular membranes (von Heijne, 1992). This in turn defines striking differences in the nitrogen content of the intracellular, transmembrane, and periplasmic domains. We have followed nitrogen allocation along membrane protein topology and we show a widespread signature of nitrogen limitation across their three different structural domains.

A parsimonious strategy of nitrogen allocation driving genetic variation in natural communities is expected to specifically target proteins that are expressed at high rate in response to nitrogen starvation. The metagenomics approach used, while relying on a thorough and widespread geographical sampling and a large amount of data, lacks the power to directly measure relative expression levels. However, insights on the stoichiometry of the proteins specifically up-regulated during nitrogen deficiency, can be gained from the well established knowledge on the metabolic response triggered by nitrogen starvation in photosynthetic bacteria. The key player is the gene NtcA, also known as the global nitrogen regulator, a transcriptional activator that triggers a signaling cascade of genes subject to nitrogen control (García-Domínguez et al., 2000; Schwarz and Forchhammer, 2005). Glutamine synthase (glnA) is then up-regulated (Tanigawa et al., 2002) to control the intracellular nitrogen flow, along with an augmented expression of the transporters involved in nitrogen uptake from the environment, such as the urtABCDE operon (Beckers et al., 2004) and the amt gene family (Paz-Yepes et al., 2008). In the opposite scenario, when nitrogen is instead particularly abundant, a completely different metabolic network is activated to exploit the abundance of this important nutrient. Photosynthetic organisms store nitrogen as arginine (the most nitrogen-rich amino acid) by relieving the feedback mechanisms of arginine biosynthesis (Llácer et al., 2008), resulting in a downstream up-regulation of the Arg gene family involved in the different steps of arginine biosynthesis (Cunin et al., 1986). We contrasted therefore the NtcA-induced cascade to the arginine biosynthetic pathway. We have used a highly reliable functional gene prediction approach based on hidden Markov models (HMMs) (Eddy, 2011) to identify these proteins in the dataset, and compared nitrogen allocation in these two metabolic networks along with the environmental nitrogen gradient from coastal regions to open oceans.

In the context of an adaptive process that preserves functional efficiency while parsimoniously allocating nitrogen, it is particularly relevant to address the role of functional and structural constraints in shaping nitrogen allocation in those two metabolic networks. To achieve this, we have used evolutionary rates as an informative proxy for functional constraints (Graur, 1985), following the rationale that sites highly constrained by structural and functional requirements will be under strong purifying selection, and tend to evolve slower than sites with a less stringent role in protein function (Kimura, 1983). After estimating the evolutionary rate of each residue in each protein using (Ashkenazy et al., 2010), we report here a clear signature of nitrogen limitation along the NtcA-induced cascade up-regulated in response to nitrogen starvation. Furthermore, we show that this signal is indeed localized in sites with lower functional constrains. As expected in the context of an adaptive and specific response to nitrogen limitation, such a pattern is not detectable in the arginine anabolic apparatus. The results presented indicate a fundamental role of selection for parsimonious nitrogen allocation in constraining protein composition in open ocean microbial communities, and strongly advocate for material costs as a key factor shaping natural selection in natural ecosystems.

## Materials and Methods

### Global Ocean Sampling Metagenome

More than 40 million assembled peptides of the Global Ocean Sampling metagenome (Rusch et al., 2007; Yooseph et al., 2007) were downloaded from the CAMERA portal (Sun et al., 2011) (April 30, 2012). Along with providing latitude and longitude, the authors have classified the sampling location as open ocean, coastal, and estuary. We have relied on these classification and worked with sequences from surface water samples with locations “open ocean,” “coastal,” and “estuary” (**Supplementary Table S1**). Estuary regions are a specific type of coastal regions where a river or a stream meets the ocean. Therefore, due to the very low number of estuary samples, they were merged with coastal samples. Only proteins longer than 100 amino acids were analyzed.

### Transmembrane Protein Topology Prediction

Membrane proteins were identified using Phobius 1.01 (Käll et al., 2004). This approach is highly sensitive, and it is based on a HMM that models the different sequence regions of a signal peptide and the different regions of a transmembrane protein in a series of interconnected states (Käll et al., 2004). Sequences with at least one transmembrane domain were classified as transmembrane proteins, and further dissected into “Cytoplasmic,” “Transmembrane,” and “Periplasmic/Extracellular” domains based on the topology prediction (signal peptides were not included in the analysis). Analyses of the three topological domains (intracellular, transmembrane, and extracellular) were performed on proteins longer than 100 amino acids and with at least 20 amino acids in each type of domain.

### HMM-Based Functional Annotation

The HMMER3 (Eddy, 2011), a software suite for protein sequence similarity searches based on probabilistic methods, was used for the functional annotation of the genes involved in the NtcA-mediated response to nitrogen scarcity, and in arginine biosynthesis. The HMMER3 package hmmscan (Eddy, 2011) was used with TIGRFAM models^1^ (Selengut et al., 2007), a collection of protein families featuring curated multiple sequence alignments, and HMMs designed to support the automated functional identification of proteins by sequence homology. For a list of genes, see **Supplementary Table S2**. Sequences were filtered based on the model-specific noise-cutoffs.

### Estimation of Evolutionary Rates

Evolutionary rates were estimated for each amino acid residue using ConSurf1.0 (Ashkenazy et al., 2010), a method based on empirical Bayesian inference. This approach explicitly accounts for the stochastic process underlying sequence evolution and takes into account their phylogenetic relationships, enabling high sensitivity in discriminating sequence conservation due to short evolutionary time from conservation due to purifying selection (Celniker et al., 2013). For each protein a set of homologs was retrieved using Swiss-Prot and UniProtKB/TrEMBL as first and second pass BLAST database, respectively. A multiple sequence alignment was performed and a phylogenetic tree reconstructed with a neighbor joining approach. Referring to the software settings, position-specific evolutionary rates where calculated based on (i) Bayesian inference, (ii) JTT substitution model, (iii) Jukes–Cantor model for the tree search, and (iv) no branch length optimization. As in the default settings, all positions that in the multiple alignment had less than 10% un-gapped amino acids were excluded from the analysis. Sites were classified based on the normalized scores (average score for all residues is 0, and the standard deviation is 1) with -0.75 as a cutoff for slow evolving sites and 0.75 for fast evolving sites.

### Nitrogen Content Analyses

Nitrogen content Nc was calculated for each sequence as follows:

$$\text{Nc}=(\Sigma n_{i})/L$$

where *n_i_* is the number of nitrogen atoms in the *i*^th^ residue side chain and *L* is the length of the sequence (*n* = 1 for asparagine, glutamine, lysine, and tryptophan; *n* = 2 for histidine; *n* = 3 for arginine; and *n* = 0 for the rest).

Plots and statistics were done with R (version 2.15), and nitrogen content calculation and sequence parsing with rpy (Python 2.7).

## Results

**FIGURE 1:**
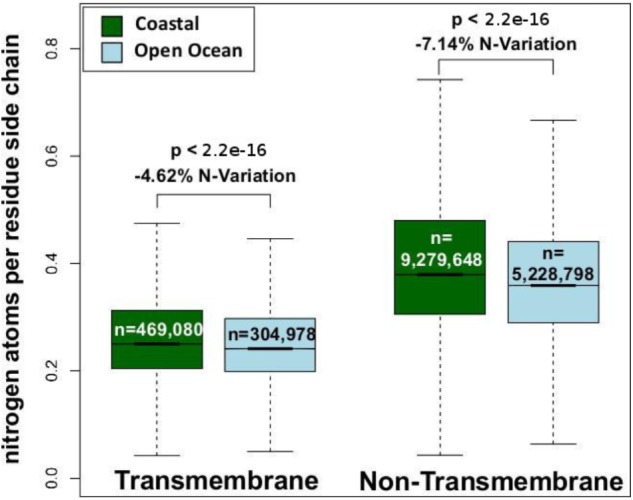
Nitrogen content of transmembrane and non-transmembrane proteins from coastal and open ocean samples: a box plot visualization. The overall variation between open ocean and coastal samples is estimated as the relative difference in mean nitrogen content. Statistical significance is based on unpaired, one-sided approximative Wilcoxon–Mann–Whitney test.

**FIGURE 2:**
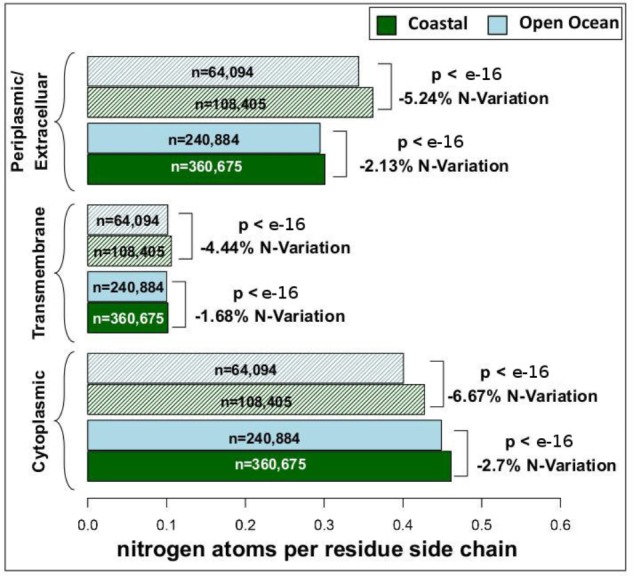
Nitrogen content and transmembrane protein topology. Mean nitrogen content of cytoplasmic, transmembrane, and periplasmic/extracellular domains from coastal and open ocean samples. Solid bars represent transmembrane proteins with more than one transmembrane domain, while striped bars represent transmembrane proteins with only one transmembrane domain. The overall variation between open ocean and coastal samples is estimated as the relative difference in mean nitrogen content. Statistical significance is based on unpaired, one-sided approximative Wilcoxon–Mann–Whitney test. Standard errors of the mean are <0.001 for all samples (thus not visible in the present plot).

We have analyzed the nitrogen content of membrane and non-membrane proteins in natural communities adapted to open ocean or coastal regions. The comparison of over 20 million sequences shows a statistically significant (one-sided Wilcoxon rank sum test) difference accounting for a decrease in nitrogen content of 4–7% in open ocean communities (**Figure 1**). After predicting protein topology for each membrane protein using (Käll et al., 2004), we have studied nitrogen allocation separately in transmembrane, intracellular, and periplasmic domains. We document here a widespread and consistent signal in each of these three structurally different domains (**Figure 2**), further reinforcing the idea of a persistent and ubiquitous signature of selection for nitrogen conservation.

In order to have a relevant impact on the total nitrogen budget, an adaptive strategy of thrifty nitrogen allocation predicts that the largest difference between open ocean and coastal communities will be in genes up-regulated in response to nitrogen limitation. Furthermore, natural selection is expected to preserve functional efficiency while parsimoniously allocating nitrogen. Using evolutionary rates as a proxy for functional constraints, we expect faster evolving sites to be the primary targets of selection for nitrogen conservation, while slow evolving sites tend to be frozen by severe functional constraints (Tourasse and Li, 2000). To test these predictions, we have narrowed the focus on the pathways that enables cells to specifically respond to critical nitrogen scarcity. We have contrasted NtcA, the critical metabolic switch up-regulated in response to nitrogen starvation, to ArgF, a key enzyme involved in the biosynthesis of arginine and down-regulated in response to nitrogen scarcity in primary producers. Using a highly reliable functional prediction approach based on high quality sequence models (see section “Materials and Methods”), we have identified over 5000 homologs for nine proteins up-regulated via NtcA in response to nitrogen scarcity (NtcA, glnA, pII, urtB, urtC, urtD, urtE, recD, and amt1) and six proteins down-regulated in response to nitrogen starvation (argC, argJ, argH, argE, argG, and argF). With a phylogenetic reconstruction approach, we have then estimated evolutionary rates for each position in each sequence in the set (see section “Materials and Methods”).

**FIGURE 3:**
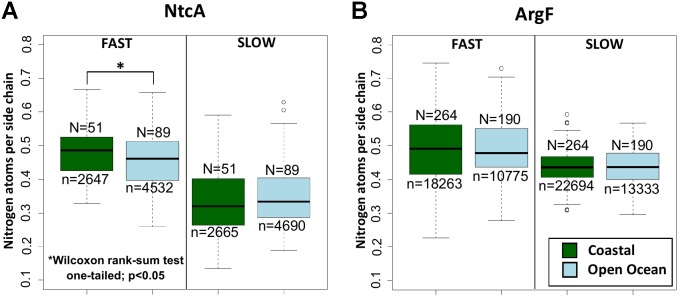
Nitrogen content allocation in NtcA and ArgF. Nitrogen content in fast and slow evolving residues (for details, see section “Materials and Methods”) in **(A)** NtcA and in **(B)** ArgF. *N* indicates the number of sequences, and *n* the number of sites in each dataset. Statistical significance is based on unpaired, one-sided Wilcoxon rank sum test.

The analysis of the 140 NtcA homologs distributed across open ocean and coastal regions indicates that the differences in nitrogen allocation are specifically localized in the fast evolving sites (**Figure 3A**), where they account for over 5% variation in nitrogen content between the two environments. Instead, the portions that are most severely constrained by functional requirements do not show any significant variation (**Figure 3A**). As expected, no significant nitrogen content variation is detectable between the natural communities adapted to coastal and open ocean regions in ArgF, neither in fast, nor in slow evolving sites (**Figure 3B**). In the present context, it is worth to note that, while different amino acids might tend to show different average evolutionary rates (Graur, 1985), the analysis performed here does not aim at comparing nitrogen composition between fast and slow evolving regions.

**FIGURE 4:**
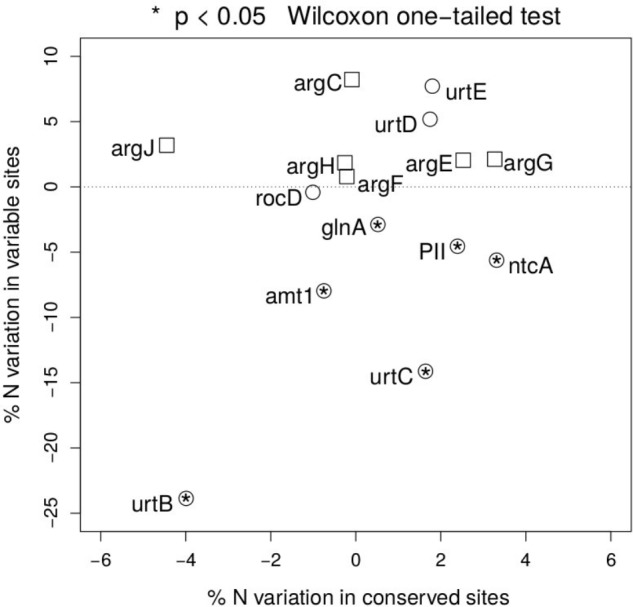
Nitrogen content of proteins up- and down-regulated in response to nitrogen depletion. Scatter plot of the variation in mean nitrogen content in open ocean relative to coastal samples in fast and slow evolving residues for the different set of proteins (circles for the NtcA-induced cascade, and squares for the arginine biosynthetic pathway). Functional identification is based on TIGRFAM model (see section “Materials and Methods”). Statistical significance is reported for variable residues, and is based on unpaired, two-sided Wilcoxon rank sum test.

To test the relevance of the results obtained beyond NtcA and ArgF, we have expanded the same approach along the two pathways, and measured the percentage nitrogen content variation between open ocean and coastal regions in fast and slow evolving sites in each gene (**Figure 4**). Following the NtcA-induced signaling cascade fired by nitrogen deprivation, we have found that, with the exception of one of the domains of the urea transporter, overall stoichiometric variation is selectively localized in fast evolving regions (**Figure 4**), where it accounts for a 5–23% statistically significant (one-sided Wilcoxon rank sum test) decrease in nitrogen content in open ocean communities. In conserved regions nitrogen content variation ranges instead between 2 and 5% (**Figure 4**.) We show a signature of nitrogen conservation in variable regions of the ammonium transporter (amt1) and in two subunits (urtB and urtC) of the ABC transporter permease proteins associated with transport of urea (Valladares et al., 2002) (**Figure 4**), while the urtD and urtE subunits do not show the same signal. Furthermore, a similar signal is detectable in the glutamine synthetase (glnA) (**Figure 4**), a key player in the condensation of inorganic nitrogen into amino acids, directly up-regulated by NtcA (Schwarz and Forchhammer, 2005). As expected, such patterns of parsimonious nitrogen allocation are not detectable along the arginine biosynthetic pathway, typically down-regulated during nitrogen scarcity (**Figure 4**), and used here as a metabolic control to the NtcA-induced pathway.

## Discussion

In microorganisms, membrane proteins are key players in the interaction between cells and their environment. Consistent with this fact, it has been shown that across marine environments membrane protein content co-varies substantially with oceanographic variables including nutrient concentration and pollution levels (Patel et al., 2010). Furthermore, due to the strong structural constraints that allow their insertion in cellular membranes, membrane proteins harbor a high percentage of hydrophobic residues, resulting in an overall low nitrogen content. Therefore, the possibility exists that the variation in the relative amount of membrane proteins alone could be a factor driving differences in the nitrogen allocation in the proteomes of natural communities adapted to open ocean and coastal environments. In order to address this potentially confounding factor we have analyzed membrane and non-membrane proteins separately, and shown a decreased nitrogen allocation in open ocean communities in both functional groups (**Figure 1**). The differences between the two environments survive and increase when addressing the role of protein structural constraints in shaping the patterns observed. Intracellular, transmembrane, and extracellular membrane protein domains separately show a statistically significant (one-sided Wilcoxon rank sum test) difference of 1–7% in nitrogen content between the two environments (**Figure 2**). This signal is particularly relevant when considering that proteins are the most nitrogen-rich component of cells, and that they constitute about 40% of the cellular dry mass (Sterner and Elser, 2002).

If evolutionary pressures for strategic nitrogen investment were a biologically relevant force able to significantly affect the cellular nitrogen budget, it is expected that they preferentially impact the stoichiometry of highly expressed proteins, especially in response to nitrogen-specific nutritional stress. Previous work on few model organisms supports this prediction, indicating that the catabolic machinery shows substantially lower N content than the anabolic machinery (Acquisti et al., 2009b). However, an understanding of the specific target of selection for parsimonious resource allocation in the genetic material of natural communities is still missing. In a regime of efficient material costs allocation, natural selection for parsimonious nitrogen usage is expected to act in synergy with other evolutionary forces, such as those that preserve the functional and structural integrity of proteins. In this case, the most effective signature of “nutrient-allocation driven” selection is expected to be located in the protein regions that are less functionally constrained.

In order to validate these predictions, we have employed a highly reliable functional prediction approach to identify proteins up- and down-regulated in response to severe nitrogen limitation in the metagenomics sequences analyzed. Furthermore, we have addressed the role of functional constraints as an alternative explanation for the pattern of nitrogen allocation observed. In the best tradition of molecular evolution (Kimura, 1983), we have used evolutionary rates as a proxy for the level of functional constraints in each protein residue. This approach has enabled us to study the variation in nitrogen allocation between natural communities adapted to open ocean and to coastal regions in more stringent evolutionary context. Finally, we have used a “metabolic control” to test the specificity of the patterns of nitrogen limitation along the NtcA-induced cascade. This was done by focusing on arginine biosynthesis, one of the key cellular routes activated by photosynthetic species to store nitrogen during nitrogen abundance. An adaptive response that shapes nitrogen material costs in proteins predicts that the NtcA-mediated pathway and the arginine anabolic pathway will show contrasting patterns of nitrogen allocation in open ocean communities. Indeed, we see that decreased nitrogen allocation is selectively localized in the functionally least constrained regions of the NtcA-induced metabolic apparatus up-regulated in response to nitrogen starvation, while it does not affect the Arg genes (**Figures 3, 4**). While the environmental data analyzed are not well suited to assess the phylogeny of different metabolic networks, it is worth to note that NtcA is known to be ubiquitous in Cyanobacteria (Frias et al., 1993). Similarly, based on KEGG database^2^, ArgE is present in the genome of marine model species in the genus *Synechococcus, Synechocystis*, and *Prochlorococcus*.

The work presented addresses the role of material costs of evolutionary change in shaping protein evolution in natural communities, taking into account the role of functional constraints. The results confirm previous observations (Grzymski and Dussaq, 2012) and indicate that the history of nitrogen availability has directly constrained the molecular architecture of the metabolic apparatus that enable cells to respond to this ecologically relevant environmental cue. Further analyses of environmental samples will be paramount in shedding further light on the mode and tempo of natural selection for parsimonious nutrient allocation in shaping the dynamics of genome evolution in natural communities.

## Author Contributions

CA designed the study, analyzed the data, and wrote the manuscript. HD performed the analyses and contributed to the writing of the manuscript. NO performed the analyses.

## Conflict of Interest Statement

The authors declare that the research was conducted in the absence of any commercial or financial relationships that could be construed as a potential conflict of interest.

## Footnotes

1 ftp://ftp.jcvi.org/pub/data/TIGRFAMs/13.0_Release/

2 https://www.genome.jp/kegg/

## Supplementary Material

TABLE S1 List of the sampling locations from the global ocean sampling metagenome.

TABLE S2 Sequences analysed in **Figure 4**: TIGRfam gene Ids, and counts.
