# Supplementary material for: Stoichio-Metagenomics of Ocean Waters: A Molecular Evolution Approach to Trace the Dynamics of Nitrogen Conservation in Natural Communities
Source: Front Microbiol. 2018 Jul 18;9:1590. doi: 10.3389/fmicb.2018.01590 (PMC6058095; doi:10.3389/fmicb.2018.01590)
Supplement: TABLE S1 — List of the sampling locations from the global ocean sampling metagenome. [file Table_1.PDF]

Table 1

GOS Id	# sequences	Site description	Region	Filter min	Filter max
GS002	435925	Coastal	North American East Coast	0.10	0.80
GS003	227142	Coastal	North American East Coast	0.10	0.80
GS004	212780	Coastal	North American East Coast	0.10	0.80
GS005	253453	Embayment	North American East Coast	0.10	0.80
GS006	202676	Estuary	North American East Coast	0.10	0.80
GS007	213665	Coastal	North American East Coast	0.10	0.80
GS008	626826	Coastal	North American East Coast	0.10	0.80
GS009	299605	Coastal	North American East Coast	0.10	0.80
GS010	303643	Coastal	North American East Coast	0.10	0.80
GS011	567170	Estuary	North American East Coast	0.10	0.80
GS012	561498	Estuary	North American East Coast	0.10	0.80
GS013	669532	Coastal	North American East Coast	0.10	0.80
GS014	483025	Coastal	North American East Coast	0.10	0.80
GS015	462767	Coastal	Caribbean Sea	0.10	0.80
GS016	475239	Coastal Sea	Caribbean Sea	0.10	0.80
GS017	927755	Open Ocean	Caribbean Sea	0.10	0.80
GS018	523739	Open Ocean	Caribbean Sea	0.10	0.80
GS019	477648	Coastal	Caribbean Sea	0.10	0.80
GS021	540820	Coastal	Eastern Tropical Pacific	0.10	0.80
GS022	427726	Open Ocean	Eastern Tropical Pacific	0.10	0.80
GS023	473053	Open Ocean	Eastern Tropical Pacific	0.10	0.80
GS026	349091	Open Ocean	Galapagos Islands	0.10	0.80
GS027	829079	Coastal	Galapagos Islands	0.10	0.80
GS028	683152	Coastal	Galapagos Islands	0.10	0.80
GS029	465644	Coastal	Galapagos Islands	0.10	0.80
GS034	574300	Coastal	Galapagos Islands	0.10	0.80
GS035	502197	Coastal	Galapagos Islands	0.10	0.80
GS036	302472	Coastal	Galapagos Islands	0.10	0.80
GS037	242864	Open Ocean	Eastern Tropical Pacific	0.10	0.80
GS047	238849	Open Ocean	Tropical South Pacific	0.10	0.80
GS049	248656	Coastal	Polynesia Archipelagos	0.10	0.80
GS109	167702	Open Ocean	Indian Ocean	0.10	0.80
GS110a	229106	Open Ocean	Indian Ocean	0.10	0.80
GS111	162527	Open Ocean	Indian Ocean	0.10	0.80
GS112a	233918	Open Ocean	Indian Ocean	0.10	0.80
GS113	308947	Open Ocean	Indian Ocean	0.10	0.80
GS114	903527	Open Ocean	Indian Ocean	0.10	0.80
GS115	175647	Open Ocean	Indian Ocean	0.10	0.80
GS116	175349	Open Ocean	Indian Ocean	0.10	0.80
GS117a	876774	Coastal sample	Indian Ocean	0.10	0.80
GS119	167470	Open Ocean	Indian Ocean	0.10	0.80
GS120	113613	Open Ocean	Indian Ocean	0.10	0.80
GS121	313296	Open Ocean	Indian Ocean	0.10	0.80
GS123	306931	Open Ocean	Indian Ocean	0.10	0.80
