# Supplementary material for: Stoichio-Metagenomics of Ocean Waters: A Molecular Evolution Approach to Trace the Dynamics of Nitrogen Conservation in Natural Communities
Source: Front Microbiol. 2018 Jul 18;9:1590. doi: 10.3389/fmicb.2018.01590 (PMC6058095; doi:10.3389/fmicb.2018.01590)
Supplement: TABLE S2 — Sequences analysed in Figure 4: TIGRfam gene Ids, and counts. [file Table_2.PDF]

Table 2

TIGRfam	Gene	# Sequences	# Amino acids analyzed
PF00543.17	PII	1344	70545
TIGR00032	argG	989	142545
TIGR00120	argJ	231	36922
TIGR00653	glnA	485	74824
TIGR00658	argF	501	65065
TIGR00836	amt1	1624	229550
TIGR00838	argH	863	133560
TIGR01850	argC	736	107097
TIGR01885	rocD	129	18871
TIGR01892	argE	81	12921
TIGR03408	urtC	98	12216
TIGR03409	urtB	53	7646
TIGR03410	urtE	108	12126
TIGR03411	urtD	81	9362
TIGR03697	ntcA	140	14534
